# The Effect of Attitude to Death on Self-Management in Patients With Type 2 Diabetes Mellitus During the COVID-19 Pandemic

**DOI:** 10.1177/00302228211020602

**Published:** 2021-06-03

**Authors:** Emine Kaplan Serin, Semra Bülbüloğlu

**Affiliations:** 1 Faculty of Health Science, Gaziantep University, Turkey; 2 Surgical Nursing Department, Erbaa Health Sciences Faculty, Gaziosmanpasa University, Erbaa Campus, Tokat, Turkey

**Keywords:** attitude to death, COVID-19, self-management, type 2 diabetes mellitus

## Abstract

This study was conducted to examine the effect of attitude to death on self-management in patients with Type 2 Diabetes Mellitus during the COVID-19 pandemic. This study was carried out in a descriptive and correlational type with the participation of n = 103 type 2 diabetes mellitus patients registered in the Internal Medicine Unit at a University Hospital. Personal Information Form, Death Attitude Profile-Revised (DAP-R), Diabetes Self-Management Questionnaire and Fear of COVID-19 Scale were used in data collection. According to the results of the study, it was determined that diabetes patients' fear of COVID-19 increased their fear of death and self-management. Similarly, neuropathy and nephropathy developed in these patients. In addition, it was determined that the diabetic patients who worked 6–7 days a week outside the home had higher levels of fear. It was found that those with high fear were more attentive to social distancing, wearing masks and hand sanitizer use. Staying at home is also not always possible for patients with chronic diseases, and people struggle with COVID-19 by working in crowded workspaces. It is necessary to recognize the struggle of patients with chronic diseases and provide social, economic and psychological support.

Achieving metabolic control of Diabetes Mellitus (DM), management of acute and chronic complications, increasing quality of life and comfort are possible with effective self-management (Hosseinzadeh et al., 2020; Pamungkas & Chamroonsawasdi, 2020). In this context, educated diabetic patients stay safer. Diabetes education is a well-accepted and effective practice that has been in the literature since ancient times. It has been actively used in clinical processes with proven positive results, and it improves self-management (Tanaka et al., 2020). Evidence that this patient group performs self-management well is the measurement results obtained to ensure optimal glycemic control (HbA1c, <7%) (Gao et al., 2020).

COVID-19 is a deadly disease emerging in Wuhan, China towards the end of 2019, and it has caused a global pandemic and continues to have its crushing effects (Pleasure et al., 2020; World Health Organization, 2020; Zhang et al., 2020). Patients with diabetes are especially hypersensitive to lung infections since their immune systems are weakened. Disease factors can be viral, bacterial and fungal pathogens. Also, the suppression of the immune system due to intensive treatment protocols makes patients susceptible to infections (Couch et al., 1997; Huang et al., 2020). The COVID-19 pandemic, which is on the rise especially in winter, gives an idea that patients with diabetes should be more protected.

Lung infections can have life-threatening or fatal consequences for people with diabetes. In the literature, it was stated that 20.3% of deaths caused by SARS-CoV-1 are patients with more than one comorbidity (Glass et al., 2004). COVID-19 leads to extremely important and terrifying consequences worldwide. Diabetic patients are at risk for COVID-19 due to a weak immune system, increased catabolism and high stress, and the course of the infection can be fatal.

Knowledge and awareness about the negative effects of COVID-19 are increasing day by day, with the impact of both press and social media. Therefore, patients with diabetes are also aware that exposure to COVID-19 can be fatal, and this may cause fear. Having a high level of fear and stress is a negative experience that gives rise to the thought of the death probability (Kapısız & Eker, 2018). Fear is a universal response to a perceived threat in people with various problems and dangers (Alvi et al., 2009). It disrupts the individual's well-being and causes emotional, physiological, and physical reactions. Fear brings about depression, anxiety and delay in wound healing in individuals and requires additional medication use (Ralph & Viljoen, 2018).

The most severe form of fear is called the fear of death (Alvi et al., 2009; Ralph & Viljoen, 2018). The physiologic balance of diabetic patients, predisposed to deteriorating, can be harmed by fear alone, even without ever being infected with the COVID-19, and this is thought to affect self-management. In this study, it was aimed to examine the effect of attitude to death on disease self-management in patients with type 2 diabetes during the COVID-19 pandemic.

## Material and Method

This study was conducted in a descriptive, cross-sectional and correlational type to examine the relationship between Type 2 Diabetes patients' attitude to death and disease self-management during the COVID-19 pandemic.

### Research Design and Participants

This study was carried out with the participation of the patients with type 2 diabetes mellitus (T2DM) who were treated in the Internal Medicine Unit and outpatient clinic of Tokat Gaziosmanpaşa University Research and Practice Hospital. This study was conducted with patients with T2DM who visited the Internal Diseases unit and outpatient clinic and accepted to participate in the study within a month after ethical approval, without using the sampling method. The data were collected by the researcher using face-to-face interviews with the patients. The data collection form was filled out by reading to the patients and marking the answers on the form by the researcher.

#### Inclusion Criteria

 i. Being diagnosed with T2DM,

ii. Being 18 years or older,

iii. Not having any communication barriers,

iv. Agreeing to participate in the study.

#### Exclusion Criteria

 i. Not being diagnosed with T2DM,

ii. Patients under the age of 18,

iii. Being diagnosed with a psychiatric disorder, non-Turkish speakers, having a communication barrier

iv. Not being willing to participate in the study.

### Data Collection Tools

In the study, “Personal Information Form”, “Death Attitude Profile-Revised”, “Diabetes Self-Management Questionnaire” and “Fear of COVID-19 Scale” were used as data collection tools. Information about the scales is presented below.

### Death Attitude Profile-Revised

The Death Attitude Profile-Revised (DAP-R) was developed by Wong (1994) to assess individuals' attitudes towards death (Wong, 1994). Its Turkish validity and reliability were performed by Işık Abalı (Işık Abalı, 2008). The scale is 32-item, multi-dimensional and Likert-type and is scored as strongly disagree (1) and strongly agree (7). There are 5 sub-scales in the scale. These are fear of death, death avoidance, neutral acceptance, approach acceptance and escape acceptance. The internal consistency reliability coefficient was 0.81 for the sum scale, 0.86 for neutral acceptance and approach acceptance subscales, 0.74 for escape acceptance, and 0.76 for fear of death and death avoidance. In this study, the Cronbach’s alpha value for the sum scale was found to be 0.91.

### Diabetes Self-Management Questionnaire

This scale is a 16-item individual assessment scale developed by Schmitt et al. in 2013 to examine the relationship of diabetic patients to diabetes self-management and glycemic control (Schmitt et al., 2013). Turkish validity and reliability were performed by Eroğlu and Sabuncu (2018). Participants were asked to answer the questions by considering their situation for the last 8 weeks. In the validity and reliability study conducted with 261 diabetic patients, the Cronbach’s alpha value was found to be 0.84.

In the study, Diabetes Self-Management Questionnaire (DSMQ) was adapted to Turkish society, and the Cronbach’s alpha value was found to be 0.85. The scale consists of 4 sub-scales.

Glucose Management: Items 1, 4, 6, 10, 12 (4th and 12th items are related to drug utilization, 1st, 6th and 10th items are related to blood glucose monitoring).

Dietary Control: Items 2, 5, 9, 13

Physical Activity: Items 8, 11, 15

Health-care Use: Items 3, 7, 14

Item 16 is not included in any sub-scale, included in sum scale only.

The rating scale was designed as a four-point Likert scale with the response options ‘applies to me very much’ (3 points), ‘applies to me to a considerable degree’ (2 points), ‘applies to me to some degree’ (1 point), and ‘does not apply to me’ (0 points).

DSMQ Scale Scoring (16 Items): The DSMQ scale consists of 16 items, and 7 of these items are formulated positively and 9 inversely. The scores of the items numbered “5, 7, 10, 11, 12, 13, 14, 15 and 16” in the scale are formulated inversely.

Scale scoring: (Total item score of the total scale or sub-scale)/(The maximum total item score that can be obtained from the total scale or sub-scale) x10). For unanswered questions, 3 points are reduced from the maximum total item score that can be obtained from the total scale or sub-scale. In the scale, a minimum of 0 and a maximum of 10 points are scored. If an item is omitted, it is evaluated as -3 points. As the points received approach 10, diabetes self-management increases. In this study, the Cronbach’s alpha value for the sum scale was found to be 0.87.

### Fear of COVID-19 Scale (FCV-19S)

It was developed by Ahorsu et al. (2020) to measure individuals' fear levels caused by COVID-19 (Ahorsu et al., 2020). The items of the scale were created based on a comprehensive review of existing scales on fear, expert evaluations, and participant interviews. The Turkish adaptation of the scale was performed by Satici et al., and the Cronbach’s alpha coefficient for internal consistency was found to be 0.84 (Satici et al., 2020).

This is a unidimensional 7-item and 5-point Likert-type scale (1 = Strongly disagree; 5 = Strongly agree). There is no reversed item on the scale. Internal consistency of the scale was found to be 0.82 and test-retest reliability was 0.72. High scores on the scale indicate that fear of COVID-19 is high. In this study, the Cronbach’s alpha value was found to be 0.90.

### Statistical Analysis

After the data were coded by the researchers, data analysis was performed by using IBM SPSS (Statistical Package for the Social Sciences) Statistics 25. Descriptive statistics were used in the analysis of the data. T-test, ANOVA analysis, correlation and regression analysis were performed to determine the relationship between scales and descriptive features. Bonferroni test was used to determine the difference in multiple comparisons. The scale reliability coefficient was determined in Cronbach's Alpha. 95% confidence interval and *p*-value less than .05 were taken into account in the evaluation of the obtained results.

### Ethics

Prior to the start of the study, the requisite legal approvals will be obtained from Tokat Gaziosmanpaşa University Ethics Committee (Decision No: 83116987–189). The Volunteer Information Form was read by the researcher by informing the patients about the research in compliance with the Declaration of Helsinki. Patients volunteering to take part in the study were included in the study after their verbal consent.

## Results

The characteristics of the diabetes patients and the mean scores obtained from the scales in this study are shown in Table 1.

**Table 1.** Characteristics of Patients With Diabetes and Mean Scores of DAP-R, DSMQ and FCV-19S (n = 103).

			DAP-R		DSMQ		FCV-19S	
Characteristics	n	%	Mean ± SD	Test	Mean±SD	Test	Mean ± SD	Test
BMI								
18.5–24.9	15	14.6	121.43 ± 4.74	F = 0.65	7.33 ± 0.43	F = 0.183	29.13 ± 2.99	F = 1.783
25–29.99	59	57.3	122.20 ± 4.31	*p* = .938	7.36 ± 0.50	*p* = .833	29.86 ± 2.53	*p* = .173
30–39.99	29	28.2	122.13 ± 4.65		7.29 ± 0.56		30.68 ± 2.89	
Gender								
Female	53	51.5	123.07 ± 4.44	t = 2.147	7.34 ± 0.45	t = 0.173	30.13 ± 2.59	t = 0.541
Male	50	48.5	121.14 ± 4.70	*p* = .034*	7.32 ± 0.57	*p* = .863	29.84 ± 2.88	*p* = .590
Marital status								
Married	92	89.3	122.58 ± 4.36	t = 2.951	7.32 ± 0.52	t=−0.589	29.78 ± 2.65	t=−2.280
Single	11	10.7	118.36 ± 5.44	*p* = .004**	7.42 ± 0.32	*p* = .557	31.72 ± 2.83	*p* = .025**
Education								
Literate	15	14.6	121.13 ± 5.04		7.29 ± 0.56		31.86 ± 2.06	
Primary school	33	32.0	122.06 ± 5.37	F = 1.258	7.36 ± 0.56	F = 1.389	30.33 ± 2.13	F = 5.688
High school	36	35.0	121.69 ± 3.77	*p* = .293	7.43 ± 0.49	*p* = .251	29.77 ± 3.09	*p* = .001**
Associate degree and above	19	18.4	123.89 ± 4.39		7.14 ± 0.36		28.31 ± 2.42	
Occupation								
Worker	24	23.3	123.50 ± 4.80		7.45 ± 0.47		28.75 ± 2.15	
Civil servant	30	29.1	121.83 ± 5.09	F = 1.993	7.33 ± 0.42	F = 0.532	28.90 ± 2.90	F = 7.991
Business owner	6	5.8	125.33 ± 4.17	*p* = .102	7.18 ± 0.34	*p* = .003**	32.66 ± 1.63	*p* = .000**
Retired	33	32	120.81 ± 3.92		7.28 ± 0.61		31.45 ± 2.13	
Unemployed	10	9.7	122.20 ± 4.41		7.33 ± 0.55		29.80 ± 2.61	
Ways of Working								
Work-from-home/home office	43	41.7	121.13 ± 4.03	F = 4.243 *p* = .007**	7.29 ± 0.59	F = 0.847 *p* = .006**	29.06 ± 2.33	F = 4.835 *p* = .003**
2 days a week outside the home	10	9.7	121.90 ± 4.62		7.20 ± 0.40		28.40 ± 2.50	
3–5 days a week outside the home	29	28.2	121.48 ± 5.20		7.34 ± 0.41		29.17 ± 2.82	
6–7 days a week outside the home	21	20.4	125.19 ± 4.09		7.48 ± 0.48		31.66 ± 2.76	
Income status								
Income < expenses	33	32.0	123.15 ± 3.94	F = 1.339	7.31 ± 0.60	F = 0.124	29.48 ± 2.70	F = 1.175
Income = expenses	44	42.7	121.90 ± 4.24	*p* = .267	7.36 ± 0.45	*p* = .884	30.02 ± 2.82	*p* = .313
Income > expenses	26	25.2	121.23 ± 5.92		7.31 ± 0.48		30.57 ± 2.56	
Smoking								
Yes	21	20.4	122.04 ± 4.96	t=−0.097	7.26 ± 0.42	t=−0.765	30.19 ± 2.63	t = 0.375
No	82	79.6	122.15 ± 4.60	*p* = .923	7.35 ± 0.53	*p* = .446	29.93 ± 2.76	*p* = .708
Alcohol consumption								
Social drinker	13	12.6	121.15 ± 5.74	t=−0.813	7.51 ± 0.37	t = 1.347	29.38 ± 2.21	t=−0.885
Addicted	90	87.4	122.27 ± 4.49	*p* = .418	7.31 ± .52	*p* = .181	30.07 ± 2.79	*p* = .394
Wearing a mask								
Yes	91	88.3	122.28 ± 4.83	t = 0.899	7.32 ± 0.50	t=−0.916	30.31 ± 2.59	t = 3.551
No	12	11.7	121.00 ± 2.82	*p* = .371	7.46 ± 0.54	*p* = .362	27.50 ± 2.54	*p* = .001**
Use of disinfectants								
Yes	89	86.4	122.28 ± 4.91	t = 0.796	7.31 ± 0.50	t=−1.278	30.30 ± 2.57	t = 3.054
No	14	13.6	121.21 ± 2.29	*p* = .428	7.50 ± 0.52	*p* = .02*	28.00 ± 2.90	*p* = .003**
Social distancing								
Yes	77	74.8	121.84 ± 4.88	t=−1.096	7.35 ± 0.51	t = 0.443	30.37 ± 2.65	t = 2.539
No	26	25.2	123.00 ± 3.83	*p* = .276	7.29 ± 0.51	*p* = .659	28.84 ± 2.66	*p* = .013**
	Mean±SD		*r*	*p*	*r*	*p*	*r*	*p*
Age	50.12 + 12.32		−.323	.001	.054	.591	.473	.000
Height	167.05 + 9.13		−.157	.113	.002	.984	−.050	.614
Weight (Kg)	79.76 + 10.16		−.108	.279	−.018	.854	.108	.277

*p < .05, **p < .01.

**F=**ANOVA; **t=**Independent sample test; **DAP-R** = Death Attitude Profile-Revised; **DSMQ** = Diabetes Self-Management Questionnaire; **FCV-19S** = Fear of COVID-19 Scale; **SD** = Standard deviation; **BMI** = Body Mass Index.

The characteristics of the patients with diabetes participating in this study are shown in Table 1. The average age of these patients was determined to be 50.12 ± 12.32. When the BMI of the patients is examined, it is seen that 57.3% of them are overweight in the range of 25–29.99. It was determined that 51.5% of the patients were female, 89.3% were married, 35% were high school graduates, 32% were retired, 41.7% were working from home every day, 42% of them had balance between their expenses and income, 79.6% did not smoke, 87.4% considered themselves as addicted to alcohol, and during the COVID-19, 88.3% wore a mask, 86.4% used disinfectant, and 74.8% practiced social distancing (Table 1).

When the mean scores obtained from the scales were examined, it was determined that the DAP-R mean score for the women was 123.07 + 4.44, for married women 122.58 + 4.36, and 125.19 + 4.09 for those who worked 6–7 days a week outside the home. It was determined that those who worked outside for 6–7 days a week had a higher fear of death than those who worked from home (*p* = .007). Similarly, it was determined that married people had a higher fear of death than singles (*p* = .004) and women had a higher fear of death than men (*p* = .034).

Considering DSMQ, self-management of civil servants and workers was found to be higher (*p* < .01). Similarly, those who work 6–7 days a week outside the home (*p* = .006) and use disinfectants (*p* = .02) were found to have higher diabetes self-management than other individuals.

When the Fear of COVID-19 Scale scores of the participants were examined, the scores of those who constantly worked from home were higher than the others (*p* = .003), and the scores of those who used masks and disinfectants and paid more attention to social distancing were found to be higher than the others (*p* < .01).

Table 2 contains information about the health status of patients with diabetes and the mean scores obtained from the scales. It was determined that the patients were followed up for an average of 16.71 ± 9.40 years with the diagnosis of diabetes, the mean HbA1C was 5.69 ± 1.31, the mean FBG was 116.27 ± 25.61 and the mean PBG was 146.33 ± 26.60. It was determined that 51.5% of diabetic patients used oral anti-diabetic drugs, 54.4% followed the diet, 70.9% did not exercise, 88.3% received training about diabetes from health professionals, and 80.6% gave regular blood. Considering the presence of complications, 5.8% had retinopathy, 14.6% had nephropathy, 20.4% had neuropathy, 12.6% had cardiovascular disease, 8.7% had cerebrovascular disease, 3.9% had diabetic foot, 11.7% had peripheral vascular disease (Table 2).

**Table 2.** Comparison of Diabetes Patients’ Health Information With the Mean Scores of the DAP-R, DSMQ and FCV-19S (n = 103).

			DAP-R		DSMQ		FCV-19S	
Health information	n	%	Mean ± SD	Test	Mean±SD	Test	Mean±SD	Test
Use of insulin								
Oral	53	51.5	122.24 ± 4.17	t = 0.244	7.33 ± 0.54	t = 0.013	30.11 ± 2.66	t = 0.469
Subcutaneous	50	48.5	122.02 ± 5.15	*p* = .807	7.33 ± 0.47	*p* = 0.989	29.86 ± 2.81	*p* = 0.640
Dietary compliance								
Yes	56	54.4	121.42 ± 4.56	t=−1.700	7.38 ± 0.49	t = 1.083	30.33 ± 2.57	t = 1.424
No	47	45.6	122.97 ± 4.66	*p* = .092	7.27 ± 0.52	*p* = 0.281	29.57 ± 2.87	*p* = 0.157
Regular exercise								
Yes	30	29.1	119.76 ± 4.62	t=−3.490	7.38 ± 0.38	t = 0.555	31.20 ± 2.63	t = 2.996
No	73	70.9	123.10 ± 4.32	*p* = .001**	7.32 ± 0.55	*p* = 0.580	29.49 ± 2.62	*p* = .003**
Diabetes education								
Yes	91	88.3	122.42 ± 4.59	t = 1.776	7.34 ± 0.52	t = 0.334	29.92 ± 2.68	t=−0.687
No	12	11.7	119.91 ± 4.66	*p* = .079	7.29 ± 0.36	*p* = .739	30.50 ± 3.11	*p* = .494
Regular blood donation								
Yes	83	80.6	122.45 ± 4.73	t = 1.438	7.35 ± 0.53	t = 0.553	29.69 ± 2.60	t=−2.253
No	20	19.4	120.80 ± 4.16	*p* = .154	7.28 ± 0.39	*p* = .581	31.20 ± 2.96	*p* = .026**
Retinopathy								
Yes	6	5.8	121.50 ± 6.68	t=−0.343	7.43 ± 0.34	t = 0.454	32.00 ± 2.82	t = 1.883
No	97	94.2	122.17 ± 4.54	*p* = .732	7.33 ± 0.52	*p* = .651	29.86 ± 2.68	*p* = .063
Nephropathy								
Yes	15	14.6	122.06 ± 6.40	t=−1.887	7.38 ± 0.30	t = 0.414	31.93 ± 2.60	t = 3.109
No	88	85.4	120.48 ± 4.23	*p* = .062	7.32 ± 0.53	*p* = .680	29.65 ± 2.62	*p* = .002**
Neuropathy								
Yes	21	20.4	122.57 ± 4.80	t=−1.744	7.39 ± 0.57	t = 0.528	31.71 ± 2.30	t = 3.411
No	82	79.6	120.53 ± 4.55	*p* = .084	7.32 ± 0.49	*p* = .599	29.54 ± 2.66	*p* = .001**
CVD								
Yes	13	12.6	123.84 ± 4.12	t = 1.425	7.35 ± 0.49	t = 0.132	30.15 ± 2.79	t = 0.230
No	90	87.4	121.88 ± 4.69	*p* = .005*	7.33 ± 0.51	*p* = .895	29.96 ± 2.77	*p* = .008**
Cerebrovascular disease								
Yes	9	8.7	122.22 ± 3.27	t = 0.058	7.06 ± 0.56	t=−1.725	30.88 ± 2.80	t = 1.035
No	94	91.3	122.12 ± 4.77	*p* = .954	7.36 ± 0.50	*p* = .088	29.90 ± 2.72	*p* = .303
Diabetic foot								
Yes	4	3.9	124.25 ± 2.98	t=−1.720	7.34 ± 0.35	t = 0.022	32.75 ± 2.21	t=−0.927
No	99	96.1	118.29 ± 4.64	*p* = .008**	7.33 ± 0.51	*p* = .982	30.04 ± 2.74	***p* = .003**
PVD								
Yes	12	11.7	122.91 ± 4.42	t = 0.617	6.89 ± 0.49	t=−3.375	29.58 ± 2.87	t=−0.548
No	91	88.3	122.03 ± 4.69	*p* = .539	7.39 ± 0.48	*p* = .001**	30.04 ± 2.71	*p* = .585
	Mean ± SD		r	*P*	r	*p*	r	*p*
Diabetes history**	16.71 ± 9.40		−.430	.000	.103	.145	.363	.000**
HbA_1_C (%)	5.69 ± 1.31		.177	.073	.096	.337	−.052	.605
FBS	116.27 ± 25.61		−.275	.005	.113	.257	.230	.020
PPG	146.33 ± 26.60		−.162	.102	.160	.107	.097	.329

**t**; independent sample t-test; **DAP-R =** Death Attitude Profile-Revised; **DSMQ =** Diabetes Self-Management Questionnaire; **FCV-19S =** Fear of COVID-19 Scale; **FBG** = Fasting blood glucose; **PBG** = Postprandial blood glucose; **PVD =** Peripheral vascular disease; **SD** = Standard deviation; *** = Displayed in years; **HbA_1_C** = Glycated hemoglobin; **CVD** = Cardiovascular disease; **SVH** = Cerebrovascular disease.

*p < .05, **p < .01.

It was determined that those who exercised regularly, those who had more diabetes history and those with neuropathy and nephropathy had higher fear of COVID-19 death (*p* < .01). Similarly, it was determined that those without PVD had better diabetes self-management (*p* < .01). In our study, both the fear of death and the fear of COVID-19 were found to be statistically higher in patients with CVD than the others (*p* < .01). The fear of COVID-19 in diabetic patients with neuropathy and nephropathy was statistically higher than the others (*p* < .01).

Table 3 contains information about the mean scores of the total and subscales of the scales. The patients scored an average of 29.99 + 2.72 points on the Fear of COVID-19 Scale. In DAP-R, the lowest score was taken from the subscales of escape acceptance (20.62 ± 2.82), the highest score from neutral acceptance and approach acceptance (53.93 ± 3.18). The mean total score of the DAP-R was 122.13 ± 4.65. The highest score on the Diabetes Self-Management Questionnaire was taken from the health-care use subscale (8.72 + 1.09), and the second highest score was taken from the glucose management subscale (7.98 ± 0.78). The mean DSMQ total score was found to be 7.33 ± 0.51.

**Table 3.** DAP-R, DSMQ and FCV-19S Score Averages (n = 103).

Total and sub-scales	Item number	Items	Score range	Min.-Max.	Mean ± SD
Fear of COVID-19 Scale (FCV-19S) Total	7	(12,34,567)	7–35	23–35	29.99 + 2.72
Neutral Acceptance and Approach Acceptance	12	(468,121,314,151,921,222,325)	12–84	44–61	53.93 ± 3.18
Escape Acceptance	5	(5,91,12,024)	5–35	14–27	20.62 ± 2.82
Fear of Death and Death Avoidance	9	(1,23,71,01,61,71,826)	9–63	40–55	47.58 ± 3.37
Death Attitude Profile-Revised (DAP-R) Total	26	(1,234,567,891,011,121,314,151,617,181,920,212,223,242,526)	26–182	110–132	122.13 ± 4.65
Glucose Management	5	(14,61,012)	0–10	6.00–9.33	7.98 ± 0.78
Dietary Control	4	(25,913)	0–10	1.67–8.33	5.70 ± 1.28
Physical Activity	3	(81,115)	0–10	3.33–8.89	6.56 ± 1.32
Health-care Use	3	(3, 7, 14)	0–10	5.56–10	8.72 ± 1.09
Diabetes Self-Management Questionnaire (DSMQ) Total	16	(12,345,678,910,111,213,141,516)	0–10	5.21–8.54	7.33 ± 0.51

**Figure 1.**
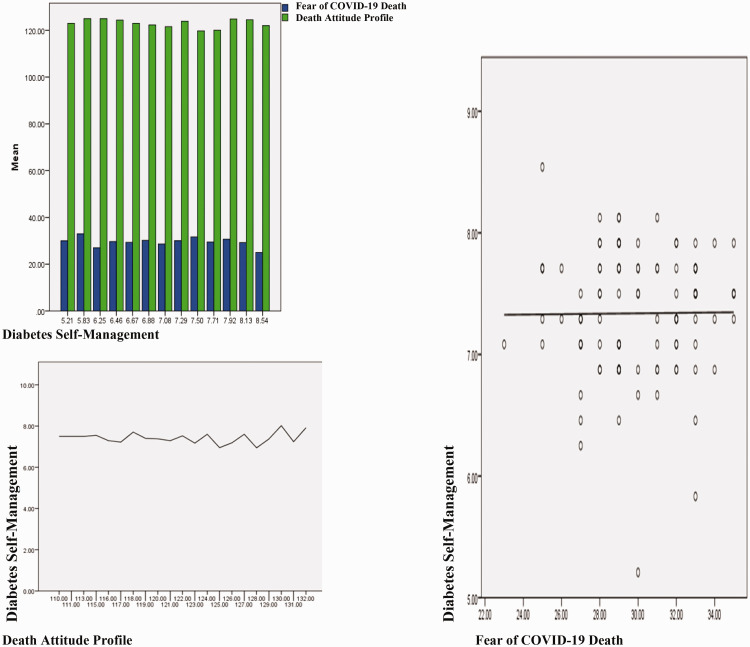
Mean Score Comparison of DAP-R, DSMQ and FCV-19S.

In Figure 1, the comparison of the total scores of the scales is schematized. Accordingly, diabetes self-management and fear of COVID-19 and attitude towards death are affected by each other. Fear of death and COVID-19 trigger each other, and diabetes self-management increases with the effect of both.

According to the regression analysis, diabetes self-management predictors are shown in Table 4. Accordingly, it is seen that the first predictor of self-management is PVD (10%) and has a significant effect (*p* < .001). It was determined that the predictors of PVD, fear of death, nephropathy, and ways of working had a significant effect on the Glucose Management subscale by 21% (*p* = .000). Nephropathy, PVD, diabetic foot and ways of working predictors affect the Dietary Control subscale by 19% (*p* < .001). The predictors of escape acceptance, Diabetic foot, DAP-R, occupation and weight affect the Physical Activity subscale by 27% (*p* < .001). The predictors of diabetes education from professional health workers, fear of death and smoking affect the Health-care Use subscale by 14% (*p* < .001).

**Table 4.** Diabetes Self-Management Predictors According to Regression Analysis (n = 103).

			Unstandardized coefficients		Standardized coefficients	Items				
		Model	B	Std. error	Beta	t	*p*	F	Sig.	R^2^
Self-management	**1**	(Constant)	30.661	1.371		22.358	.000	11.388	.001^a^	.101
		PVD*	2.422	.718	.318	3.375	.001			
Glucose Management	1	(Constant)	6.830	.445		15.333	.000	6.939	.010^a^	.064
		PVD	.614	.233	.254	2.634	.010			
	2	(Constant)	9.295	1.163		7.992	.000	6.228	.003^b^	.111
		PVD	.572	.229	.236	2.494	.014			
		Fear of Death	−.050	.022	−.216	−2.286	.024			
	3	(Constant)	8.819	1.139		7.743	.000	6.996	.000^c^	.175
		PVD	.601	.222	.248	2.704	.008			
		Fear of Death	−.064	.022	−.275	−2.922	.004			
		Nephropathy	.574	.207	.260	2.775	.007			
	4	(Constant)	9.021	1.123		8.030	.000	6.553	.000^d^	.211
		PVD	.625	.219	.258	2.861	.005			
		Fear of Death	−.076	.022	−.330	−3.438	.001			
		Nephropathy	.612	.204	.278	3.001	.003			
		Ways of working	.128	.061	.198	2.118	.037			
Dietary Control	1	(Constant)	7.432	.657		11.319	.000	7.177	.009^e^	.066
		Nephropathy	−.932	.348	−.258	−2.679	.009			
	2	(Constant)	5.656	.984		5.746	.000	6.588	.002^f^	.116
		Nephropathy	−.880	.341	−.243	−2.582	.011			
		PVD	.892	.375	.224	2.381	.019			
	3	(Constant)	2.916	1.549		1.882	.063	6.275	.001^g^	.160
		Nephropathy	−.921	.334	−.255	−2.755	.007			
		PVD	.950	.368	.239	2.580	.011			
		t	1.381	.611	.209	2.260	.026			
	4	(Constant)	2.443	1.543		1.583	.117	5.879	.000^h^	.194
		Nephropathy	−.903	.329	−.250	−2.740	.007			
		PVD	.999	.363	.251	2.751	.007			
		Diabetic foot	1.330	.602	.201	2.208	.030			
		Ways of working	.196	.097	.184	2.025	.046			
Physical Activity	1	(Constant)	9.198	.932		9.872	.000	8.107	.005^ı^	.074
		Escape acceptance	−.127	.045	−.273	−2.847	.005			
	2	(Constant)	12.284	1.550		7.925	.000	7.278	.001^i^	.127
		Escape acceptance	−.128	.044	−.275	−2.940	.004			
		Diabetic foot	−1.563	.636	−.230	−2.459	.016			
	3	(Constant)	5.470	3.251		1.683	.096	6.947	.000^j^	.174
		Escape acceptance	−.153	.044	−.327	−3.482	.001			
		Diabetic foot	−1.826	.631	−.268	−2.892	.005			
		DAP-R**	.064	.027	.226	2.369	.020			
	4	(Constant)	3.582	3.225		1.111	.269	7.415	.000^l^	.232
		Escape acceptance	−.176	.043	−.376	−4.052	.000			
		Diabetic foot	−1.825	.612	−.268	−2.982	.004			
		DAP-R	.078	.027	.275	2.920	.004			
		Occupation	.239	.088	.249	2.731	.007			
	5	(Constant)	.809	3.381		.239	.811	7.240	.000^k^	.272
		Escape acceptance	−.192	.043	−.410	−4.452	.000			
		Diabetic foot	−1.975	.603	−.290	−3.277	.001			
		DAP-R	.088	.027	.311	3.324	.001			
		Occupation	.254	.086	.265	2.955	.004			
		Weight	.026	.012	.204	2.291	.024			
Health-care Use	1	(Constant)	9.576	.382		25.058	.000	5.339	.023^m^	.050
		Diabetes Education	−.760	.329	−.224	−2.311	.023			
	2	(Constant)	13.066	1.537		8.503	.000	5.529	.005^n^	.100
		Diabetes Education	−.808	.323	−.238	−2.504	.014			
		Fear of Death	−.072	.031	−.223	−2.341	.021			
	3	(Constant)	12.358	1.536		8.045	.000	5.599	.001^o^	.145
		Diabetes Education	−.890	.318	−.262	−2.801	.006			
		Fear of Death	−.077	.030	−.238	−2.554	.012			
		Smoking	.581	.253	.215	2.295	.024			

^a^Predictors: (Constant), PVD.

^b^Predictors: (Constant), PVD, Fear of Death.

^c^Predictors: (Constant), PVD, Fear of Death, Nephropathy.

^d^Predictors: (Constant), PVD, Fear of Deat,. Nephropathy, Ways of working.

^e^Predictors: (Constant), Nephropathy.

^f^Predictors: (Constant), Nephropathy, PVD.

^g^Predictors: (Constant), Nephropathy, PVD, Diabetic foot.

^h^Predictors: (Constant), Nephropathy, PVD, Diabetic foot, Ways of working.

^ı^Predictors: (Constant), Escape acceptance.

^i^Predictors: (Constant), Escape acceptance, Diabetic foot.

^j^Predictors: (Constant), Escape acceptance, Diabetic foot, DAP-R.

^k^Predictors: (Constant), Escape acceptance, Diabetic foot, DAP-R, Occupation, Weight.

^l^Predictors: (Constant), Escape acceptance, Diabetic foot, DAP-R, Occupation.

^m^Predictors: (Constant), Diabetes Education.

^n^Predictors: (Constant), Diabetes Education, Fear of Death.

^o^Predictors: (Constant), Diabetes Education, Fear of Death, Smoking.

*PVD: Peripheral vascular disease.

**DAP-R: Death Attitude Profile-Revised.

## Discussion

People with diabetes feel the negative effects of the COVID-19 process at a high level. The risk of infection is high in this patient group, and they are in the category of patients with chronic diseases. People with diabetes have impaired phagocytosis, bactericidal activity, impaired neutrophil chemotaxis and impaired innate cell-mediated immunity (Ma & Holt, 2020). The COVID-19, which directly threatens the immune system and is associated primarily with the cause of death in chronic diseases, has become a higher rate of death in diabetic patients than cardiovascular diseases by causing severe pneumonia (Wu et al., 2020).

COVID-19 has a 4.6% mortality rate worldwide (World Health Organization, 2020). It has been determined that 10.5% of these deaths are due to Cardiovascular Diseases (CVD) (Wu et al., 2020). In contrast, diabetes was common comorbidity in SARS-CoV-1 and MERS (Middle East Respiratory Syndrome) patients prior to COVID-19. In SARS, the prevalence of Diabetes Mellitus (DM) was 11%, and the presence of diabetes was thought to increase the risk of death by twelvefold (Booth et al., 2003; Chan et al., 2003). DM and hypertension were found in 50% of MERS cases (Badawi & Ryoo, 2016). In addition, it has been determined that patients with hypertension and DM have a high risk of developing CVD caused by COVID-19 and the mortality rate in these patients is more than 50% (Yang et al., 2020; Zheng et al., 2020; Zhou et al., 2020). In a previous study, it was found that 16% of severe COVID-19 cases and 5.7% of mild ones were patients with diabetes (Guan et al., 2020).

In our study, the rates of patients with CVD, neuropathy and nephropathy were 12.6%, 20.4% and 14.6%, respectively. Both the fear of death and the fear of COVID-19 were found to be high in patients with CVD (*p* < .01). In addition, diabetic patients with neuropathy and nephropathy were found to have a higher fear of COVID-19 than others (*p* < .01). When the types of fear felt by diabetic patients in the literature were examined, it was determined that amputation, hypoglycemic coma, blindness, and fear of disease progression were found to be the most common in patients aged 60 and over (Quandt et al., 2013). In the same study, it was found that the rate of those with fear of amputation was 33%, and those who feared blindness were 25% (Quandt et al., 2013). Another study found that patients with type 2 diabetes were afraid of having retinopathy, amputation, nephropathy, neuropathy, and stroke (Hendricks & Hendricks, 1998). In our study, the rate of patients with the diabetic foot was determined as 3.9%. In addition, the fear of death and COVID-19 in diabetic foot patients was found to be higher than the others (*p* < .01). In a previous study, it was stated that the greatest fear of patients with diabetic foot complications was death (Wukich et al., 2018).

The continuity of the problems caused by diabetes is due to the chronicity of the disease. Self-management plays a key role in the effective treatment of diabetes. Facilitating and preventing factors determine the effectiveness of self-management in DM (Kristianingrum et al., 2018). While family, social support and healthcare workers’ support are among the facilitating factors (Shen et al., 2013), the COVID-19 pandemic can be evaluated in the category of preventing factors. Indeed, in our study, it was found that civil servants and workers had higher diabetes self-management than other occupational groups, and it was determined that those who work 6–7 days a week outside the home have higher diabetes self-management than those who work from home or less outside. In addition, when looking at the Fear of COVID-19 scale scores of diabetic patients, it was found that those who constantly work from home have lower scores than others (*p* = .003), and it was also found that those wearing masks and using disinfectants and those who pay more attention to social distance have higher scores than the others (*p* < .01).

It is thought that there is an inverse relationship between good self-management and the duration of the disease in patients with diabetes. It is not always possible to cover the costs in terms of medicine, diet and hygiene products, which leads to the material and moral burnout of diabetic patients over the years. In a previous study, it was reported that the self-management of individuals diagnosed with DM for more than 5 years was impaired (Adwan & Najjar, 2013). Neuropathy, nephropathy and retinopathy, which are long-term complications of diabetes, have been associated with impaired self-management and poor compliance (Adwan & Najjar, 2013).

In our study, predictors of diabetes self-management obtained from regression analysis such as PVD, fear of death, nephropathy and ways of working were found to be statistically significant, and they were effective between 10% and 27% (*p* < .01). It was determined that our participants had an average diabetes history of 16.71 ± 9.40 years. Those adapting to the diet were 54.4%, those having diabetes education were 88.3%, and those giving regular blood for routine health check-ups were determined as 80.6%. It is stated in the literature that the support of healthcare professionals, family and relatives is very important in diabetes self-management (Chlebowy et al., 2010). In our study, it was found that a high score of 7.33 ± 0.51 (5.21–8.54) was obtained from Health-care Use, which is one of the subscales of DSMQ. The results obtained from our study are similar to the literature. Future studies can address the self-management of the elderly patient group, whose self-care potential is weakened and complications are severe.

## Conclusion

In individuals with diabetes, the fear of COVID-19 increases the fear of death, and self-management is seen as an effective plan/method to reduce the risk of death. As a result of this study, the fear of death, which was already a risk and cause of unhappiness for patients with diabetes before the pandemic, became more severe during the pandemic, and the risk of COVID-19 transmission made life even more stressful.

Given the uncertainty of COVID-19 vaccines and available treatments, the main strategy to fight against the pandemic is social distancing, hand hygiene and wearing masks. The impact of the pandemic on social interactions, patients with chronic diseases, healthcare and the economy has been increasing. Staying at home is also not always possible for patients with chronic diseases, and people struggle with COVID-19 by working in crowded workspaces. It is very important to recognize the struggle of patients with chronic diseases, to provide social, economic and psychological support, and to integrate the findings of research like this into daily life.
